# Gene gain and loss from the Asian corn borer W chromosome

**DOI:** 10.1186/s12915-024-01902-4

**Published:** 2024-05-01

**Authors:** Wenting Dai, Judith E. Mank, Liping Ban

**Affiliations:** 1 Department of Grassland Resources and Ecology, College of Grassland Science and Technology, China Agricultural University, Beijing, 100193 China; 2 Department of Zoology and Biodiversity Research Centre, University of British Columbia, Vancouver, V6T 1Z4 Canada

**Keywords:** *Ostrinia furnacalis*, Genome assembly, Sex chromosome

## Abstract

**Background:**

Sex-limited chromosomes Y and W share some characteristics, including the degeneration of protein-coding genes, enrichment of repetitive elements, and heterochromatin. However, although many studies have suggested that Y chromosomes retain genes related to male function, far less is known about W chromosomes and whether they retain genes related to female-specific function.

**Results:**

Here, we built a chromosome-level genome assembly of the Asian corn borer, *Ostrinia furnacalis* Guenée (Lepidoptera: Crambidae, Pyraloidea), an economically important pest in corn, from a female, including both the Z and W chromosome. Despite deep conservation of the Z chromosome across Lepidoptera, our chromosome-level W assembly reveals little conservation with available W chromosome sequence in related species or with the Z chromosome, consistent with a non-canonical origin of the W chromosome. The W chromosome has accumulated significant repetitive elements and experienced rapid gene gain from the remainder of the genome, with most genes exhibiting pseudogenization after duplication to the W. The genes that retain significant expression are largely enriched for functions in DNA recombination, the nucleosome, chromatin, and DNA binding, likely related to meiotic and mitotic processes within the female gonad.

**Conclusions:**

Overall, our chromosome-level genome assembly supports the non-canonical origin of the W chromosome in *O. furnacalis,* which experienced rapid gene gain and loss, with the retention of genes related to female-specific function.

**Supplementary Information:**

The online version contains supplementary material available at 10.1186/s12915-024-01902-4.

## Background

The non-recombining, sex-specific portions of the genome, namely Y and W chromosomes, exhibit very different properties from the remainder of the genome [1]. Recent advances in sequencing have made it possible to assemble full, or nearly full, sequences of numerous Y chromosomes [2], and these efforts have revealed general patterns such as the retention of genes related to dosage-sensitivity [3, 4] and male function [5–8] as one might expect from a chromosome limited in its inheritance to males.

Studying Y chromosomes in male heterogametic systems alone makes it difficult to differentiate the effects of sex limitation from the effects of limitation to males, and so W chromosomes in female heterogametic organisms can be a powerful contrast to reveal the overall convergence of genomic patterns of these unusual regions of the genome [9]. Despite providing an important comparison, sequencing of W chromosomes has lagged somewhat, with more and more complete assemblies in Lepidoptera suggesting that W chromosomes share some similarities with Y chromosomes, namely an abundance of repetitive elements [10, 11]. However, some evidence from birds suggests that W chromosomes may differ from Y chromosomes in that they lack genes with female-specific functions [3, 12]. Whether this is unique to birds, or is a broader pattern of W chromosomes, remains unclear.

Lepidoptera, butterflies and moths, provide a key additional female heterogametic system [13, 14]. The conservation of the Z chromosome has been well established in Lepidoptera [15]; however, the W chromosome in Lepidoptera is unusual in that it was recruited into the genome well after the origin of the Z chromosome [16], as the basal lineages in the clade are Z0/ZZ. Available evidence suggests that, at least in some lineages, the W chromosome bears no homology to the Z [11, 15, 17] and may actually have originated from a B chromosome [15]. Within the Lepidoptera, complex sex chromosomes including neo-W chromosomes are observed in many clades based on cytogenetic analysis [16].

Recently, third-generation sequencing advances have permitted partial or chromosome-level assemblies of the W chromosome in a limited number of Lepidoptera, including *Cydia pomonella* (Torticidae, Tortricoideae) [17], *Cnaphalocrocis medinalis* (Crambidae, Pyraloidea) [18], *Spodoptera exigua* (Noctuidae, Noctuoidea) [19], *S. frugiperda* (Noctuidae, Noctuoidea) [20], *Trichoplusia ni* (Noctuidae, Noctuoidea) [21], *Pieris mannii* (Pieridae, Papilionoidea) [10], and *Dryas iulia* (Nymphalidae, Papilionoidea) [11]. This work has revealed no detectible homology between the Z and W in *C. pomonella* [17], *T. ni* [22], and *D. iulia* [11]. In contrast, substantial synteny is observed between the W chromosomes of *S. exigua* and *C. medinalis* [18, 23]. All this is consistent with a non-canonical origin of the W chromosome in Lepidoptera, where the W has been recruited from a B chromosome and is therefore not homologous to the deeply conserved corresponding Z chromosome.

To answer questions about the conservation and gene content of the lepidopteran W, we built a chromosome-level genome of the Asian corn borer *O. furnacalis* Guenée (Lepidoptera: Crambidae, Pyraloidea), a major insect pest of corn, widespread in the Asian-Western Pacific region, using a combination of PacBio HiFi circular consensus sequencing (CCS) mode and high-throughput chromosome conformation capture (Hi-C) sequencing. We couple this with extensive RNA-Seq analysis of multiple developmental stages and tissues in both sexes. Our catalog of W gene content reveals extensive duplications from all other chromosomes in the genome, with a relatively low percent of genes with persistent gene activity, which are enriched for functions in DNA recombination, the nucleosome, chromatin, and DNA binding. Our results suggest that the W chromosome retains gene related to meiotic and mitotic functions within the female gonad, in contrast to the avian W chromosome [3, 12] and more consistent with findings from Y chromosomes [5–8].

## Results

### Chromosome-level genome assembly of the Asian corn borer

Our chromosome assembly strategy employed PacBio HiFi (CCS) sequencing data to assemble the draft genome and Hi-C data to detect chromosomal contact information. These PacBio long-reads were self-corrected using Quiver and assembled into a draft genome assembly with a total length of 493.10 Mb, consisting of 57 contigs with an N50 length of 15.69 Mb (Additional File 1: Table S1). The assembled genome size is similar to that obtained from genome surveys (Additional File 1: Figure S1). Next, Hi-C linking information further anchored, ordered, and oriented 46 contigs to 32 chromosomes (30 autosomes, with Z and W sex chromosomes), which contained 86.79% of the whole genome assembly (Additional File 1: Table S1, Figure S2). The chromosome-level genome assembly was 492.57Mb with a scaffold N50 of 16.47 Mb (Additional File 1: Table S1).

We evaluated the completeness of *O. furnacalis* genome assembly with the Benchmark of Universal Single-Copy Orthologs (BUSCO v5) from the lepidoptera_odb10 set. Our assembly contained 98.7% of complete BUSCO genes, of which 98.5% were single copy and 0.2% were duplicates (Additional File 1: Table S2). To further evaluate the genome assembly quality, genomic DNA sequencing data from Illumina HiSeq were mapped to the assembly scaffolds, with a 99.07% coverage rate (Additional File 1: Table S2). Finally, lepidopteran genomes typically exhibit very high levels of synteny. Whole-genome alignment of the *O. furnacalis* assembly to the chromosomes of the *S. litura* revealed that chromosomal linkage and ordering of genes are highly conserved (Additional File 1: Figure S3). All these analyses indicated that the genome assembly is both highly reliable and complete for subsequent analyses.

### Genome annotation

**Fig. 1:**
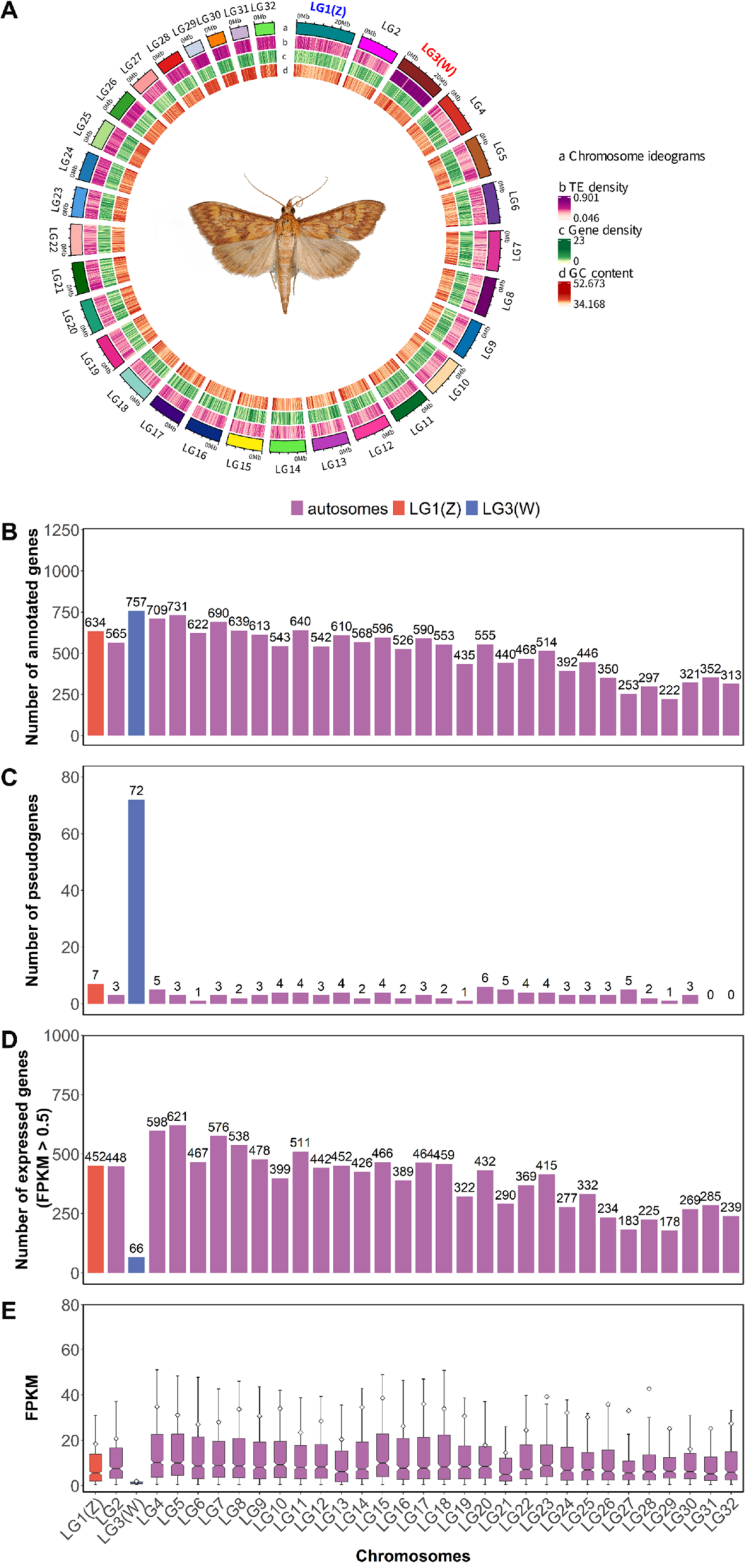
Genomic characterization of *Ostrinia furnacalis*. **A** Circos plot depicting the genomic landscape of the 32 chromosomes (Chr1–Chr32 on a Mb scale). The denotation of each track is listed in the center of the circle. Blue lines in LG3 (the W chromosome) represent collinearity within the W, due to repeat elements.** B** Number and distribution of protein-coding genes. **C** Number and distribution of pseudogenes. **D.** Number of expressed genes (FPKM > 0.5 in mixed sample). **E** Average chromosomal expression level (excluding genes with FPKM ≤ 0.5 in mixed sample)

In total, 860,391‬ repeat sequences spanning 204.4 Mb were identified, constituting 41.45% of the *O. furnacalis* genome (Additional File 1: Table S1, Fig. 1A). We integrated the result of ab initio, homology-based, and RNA-seq methods to annotate protein-coding genes, most of which came from homology-based and RNA-seq methods, indicating the high-quality of annotation (Additional File 1: Figure S4). Finally, we obtained 16,509 protein coding genes in the *O. furnacalis* genome, which has similar gene features to other lepidopteran genomes (gene length, gene number, coding length, intron length of each gene, exon length) (Additional File 1: Figure S4). Next, we identified different types of noncoding RNAs (ncRNAs), including 7710 transfer RNAs (tRNA), 73 ribosomal RNAs (rRNA), and 39 microRNAs (miRNA) (Additional File 1: Table S3). In addition, we annotated 167 pseudogenes in the *O. furnacalis* genome, defined as any genomic sequence that is similar to another gene but is defective, such as containing a premature stop codon or a frameshifts mutation [24]. Most pseudogenes were located in the chromosome LG3 (Fig 1C, Additional File 1: Table S3), the W chromosome (see below). We performed a functional annotation for all predicted protein coding genes using NCBI nonredundant, EggNOG, GO, KEGG, SWISS-PROT, and Pfam databases, with 98.21% of our predicted genes assigned to at least one of these databases (Additional File 1: Table S3).

**Fig. 2:**
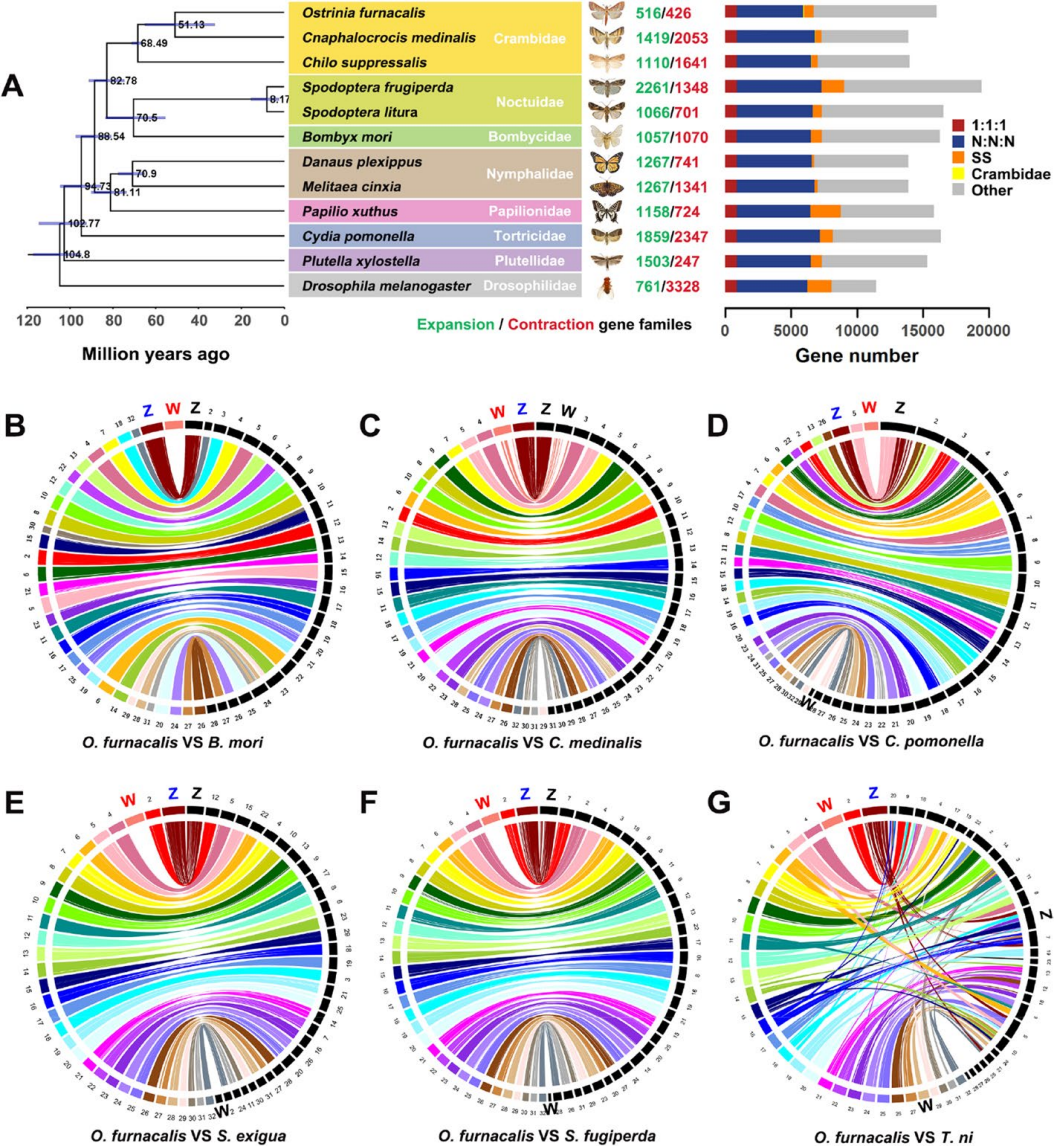
Phylogenetic tree and chromosome-level synteny analysis. **A** Phylogenetic tree and gene orthology of *Ostrinia furnacalis* with ten lepidopteran genomes and *Drosophila melanogaster*. 1:1:1 indicates universal single-copy genes, shared by 12 species with 1 copy. N:N:N indicates other universal genes. SS indicates species-specific single-copy genes. Crambidae indicates universal genes limited in Crambidae. Others indicates all other orthologous groups. Comparative analysis of synteny between *O. furnacalis* and **B** *Bombyx mori* (no assembled W), **C** *Cnaphalocrocis medinalis*, **D** *Cydia pomonella*,** E** *Spodoptera exigua, F* *Spodoptera fugiperda*, and **G** *Trichoplusia ni*. The chromosomes of *O. furnacalis* are shown in the left, and the other insects’ chromosomes are shown in the right

We used OrthoFinder to find orthologous genes among *O. furnacalis* and ten other insect species (see “ Methods”). A total of 16,364 orthologous groups with 857 single-copy orthologous genes were identified. We inferred a phylogenetic tree and divergence time estimate using the end-to-end concatenated amino acid sequences of 857 single-copy orthologous genes. Divergence time estimation indicated that the Crambidae lineage which *O. furnacalis* belongs to arose approximately 68.49 Mya ago (Fig. 2A).

### Synteny, karyotype evolution, and sex chromosomes

**Fig. 3:**
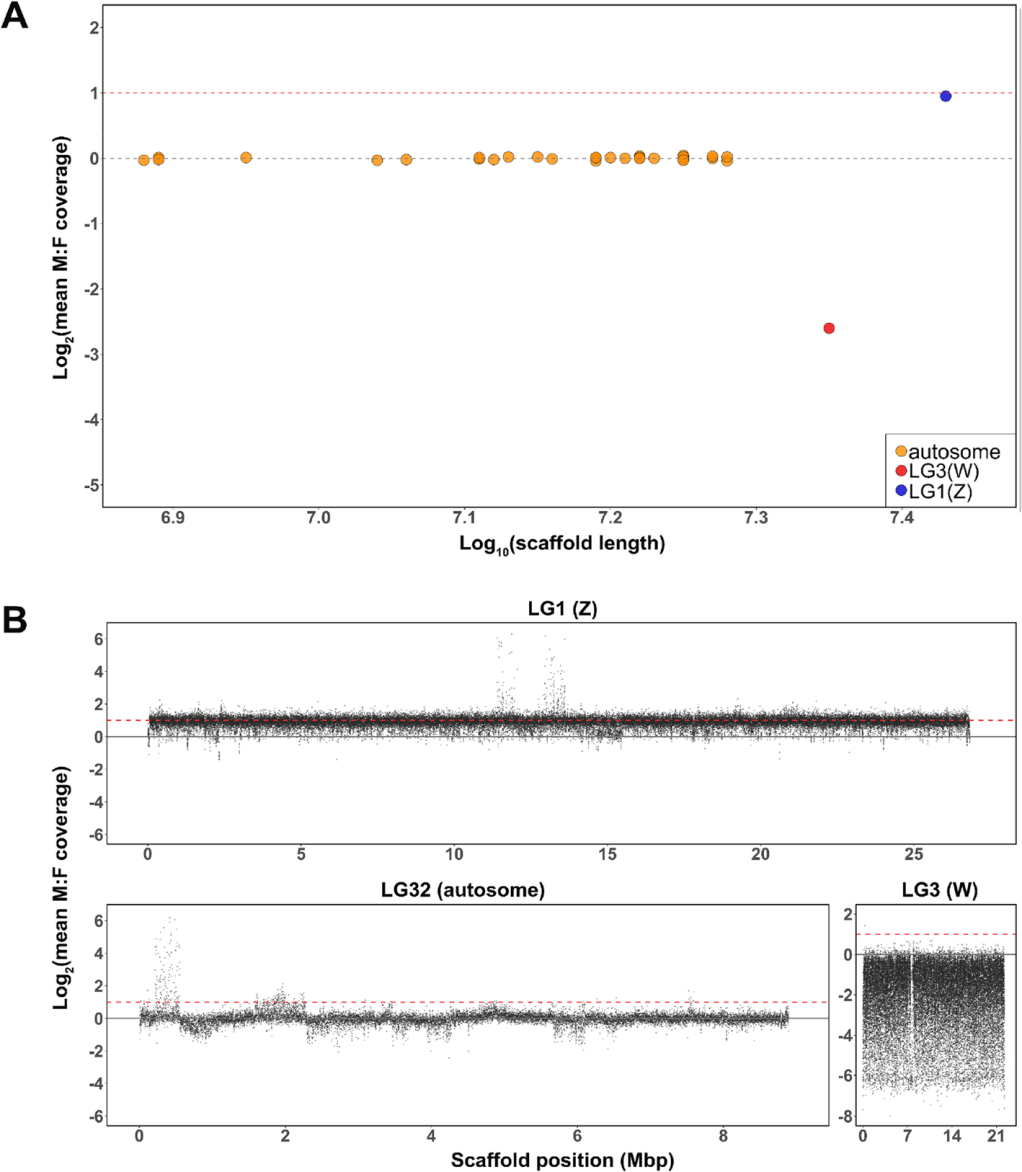
Detection of sex chromosomes.** A** Male:female coverage ratios for each chromosome, plotted by chromosome length. Each point represents a single chromosome. The dashed gray line is the theoretical expectation for autosomes and the dashed red line shows the expectation for the Z chromosome. **B** Male:female coverage ratios plotted in 500 bp windows across scaffolds for LG1(Z), LG3(W), and a representative autosome (LG32)

We compared the syntenic relationships between *O. furnacalis* and other lepidopterans*,* including *Bombyx mori, C. medinalis*, *C. pomonella*, *S. exigua, S. fugiperda*, and *T. ni* (Fig. 2B–G)*.* In general*,* the *O. furnacalis* genome shows a high level of synteny with other lepidopteran genomes, though with some fusion and fission events. *O. furnacalis* LG1 is syntenic with the Z chromosome in all other species, suggesting it is the Z chromosome in *O. furnacalis* as well, and this is also evident from M:F coverage analysis (Fig. 3A), which reveals twofold greater coverage in males (ZZ) compared to females (ZW).

*O. furnacalis* LG3, the W chromosome, exhibits female-biased coverage consistent with a female-limited chromosome (Fig. 3A). The M:F coverage is highly variable across chromosome LG3 compared to all other chromosomes (Fig. 3B), due in large part to the abundance of TEs on this chromosome (Additional File 1: Figure S5- S6). The W chromosome (LG3) is the second largest chromosome (22.23 Mb) in our assembly (Fig. 1A), with the largest number of predicted protein coding genes (excluding pseudogenes) compared with other chromosomes (Fig. 1B). The W also has the largest number of pseudogenes (Fig. 1C) and contains 43.1% of all pseudogenes annotated in the genome. Many genes that are not technically pseudogenized were expressed below our 0.5 FPKM threshold. Only 66 protein coding genes showed significant expression above this threshold in our mixed sample (comprised of eggs, larvae, pupae, and adults, see “Methods,” Fig. 1D–E); 48 W genes showed expression >0.5 FPKM in adult female gonads.

Furthermore, LG3 has a notably greater repeat density and significantly different repeat composition compared to other chromosomes (Additional File 1: Figure S5-S6) with a larger proportion of satellites, DIRS, LINE, Copia, and Gypsy, and an enrichment of *Maverick* elements.

### W homology

Given that previous reports have found no evidence of homology between the Z and W in some lepidopteran species [11, 17, 22], we next investigated the evolutionary history of the *O. furnacalis* W chromosome. Using our W gene set, we first examined homology between the *O. furnacalis* W chromosome and the W chromosome from other lepidopterans. The *O. furnacalis* W shows substantial homology with the W in *C. medinalis*, consistent with some form of shared ancestry within the family Crambidae. However, we observed no discernable homology between the *O. furnacalis* W and the W chromosome of any of the more distantly related lepidopterans that we queried (Fig. 2B–G).

**Fig. 4:**
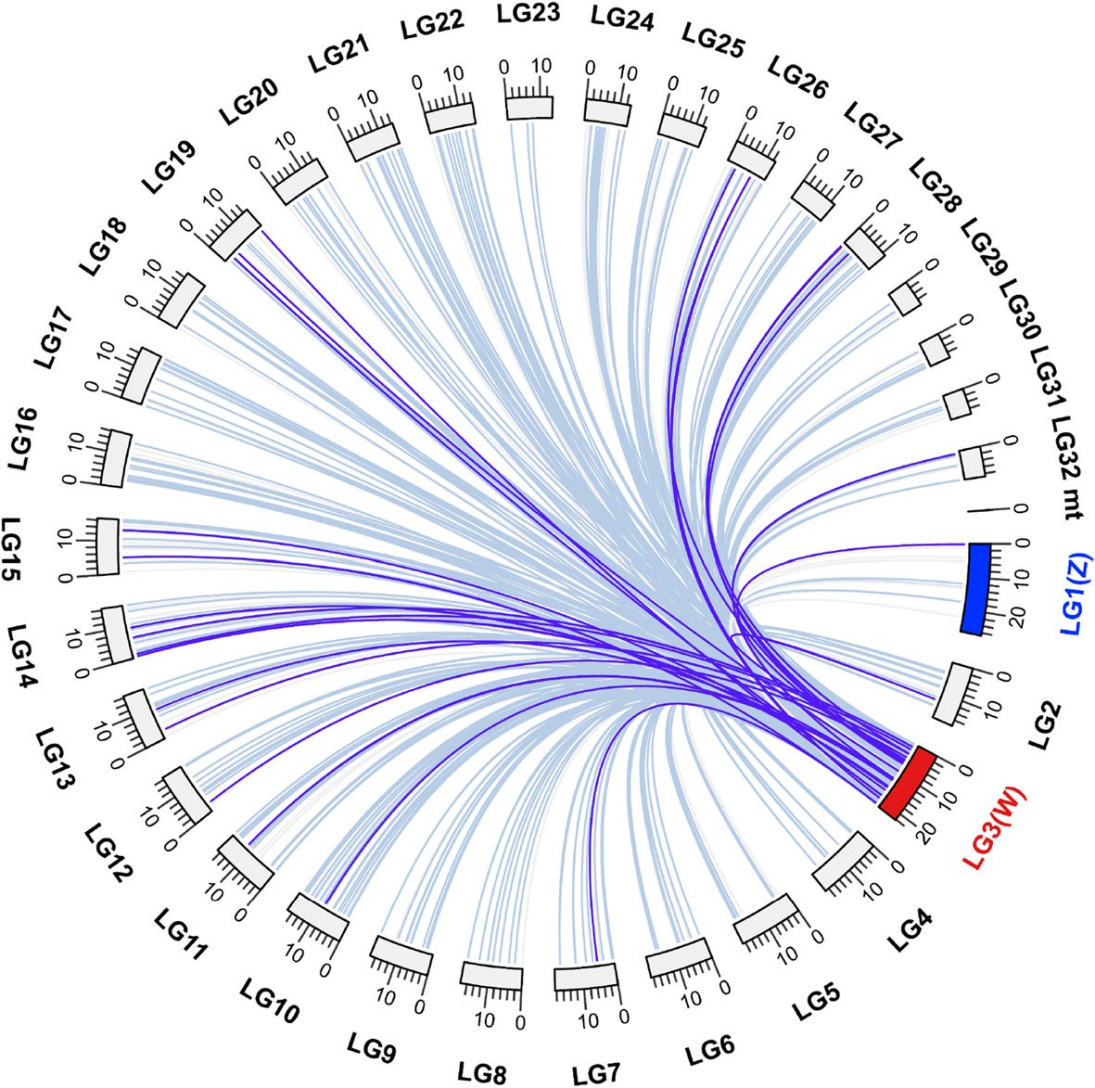
The reciprocal best hits search for W (LG3) genes. W chromosome (LG3) is marked in red and Z chromosome (LG1) in blue, mt represent the mitochondrial genome of *Ostrinia furnacalis.* Light blue line: identity ≥ 90%; purple line: 80% ≤ identity < 90%; light grey line: identity < 80%

Reciprocal best BLAST hits between the W coding catalog and the remainder of the *O. furnacalis* genome reveals similarity throughout the genome (Fig. 4), consistent with a B chromosome origin of the W chromosome followed by gene duplication from locations throughout the genome. Of the 482 W coding genes having Z/autosome paralogs (Fig. 4), 202 exhibited fewer or no introns in the W paralogs, consistent with retrotransposition.

**Fig. 5:**
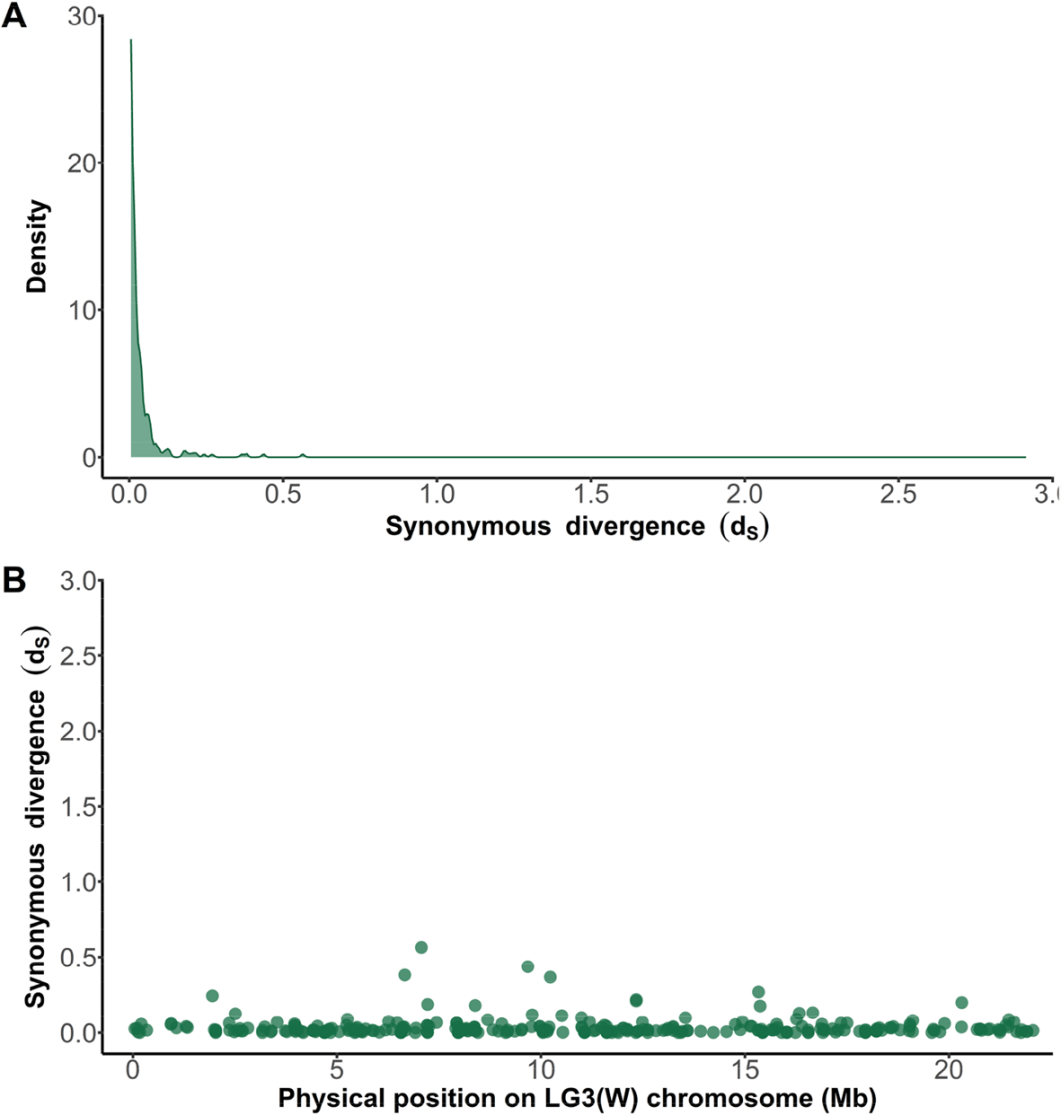
Synonymous divergence (dS) between W gene and best BLAST hit gene from the remainder of the genome. **A** Density plot of dS values. **B** The distribution of dS values along the W chromosome

We next examined synonymous divergence (dS) for these paralogs in order to determine the timing of gene duplication to the W chromosome. The density of dS (Fig. 5A) shows that most paralogs with W genes have relatively low divergence, and the distribution of paralog dS (Fig. 5B) across the W chromosome suggests that duplications occur randomly throughout the chromosome. Given the low percent of genes on the W chromosome that exhibit expression >0.5 FPKM (Fig. 1C) and the high number of pseudogenes on the W (Fig. 1D), it is likely that most duplicates to the W chromosome fail to maintain significant expression and are silenced relatively quickly.

**Fig. 6:**
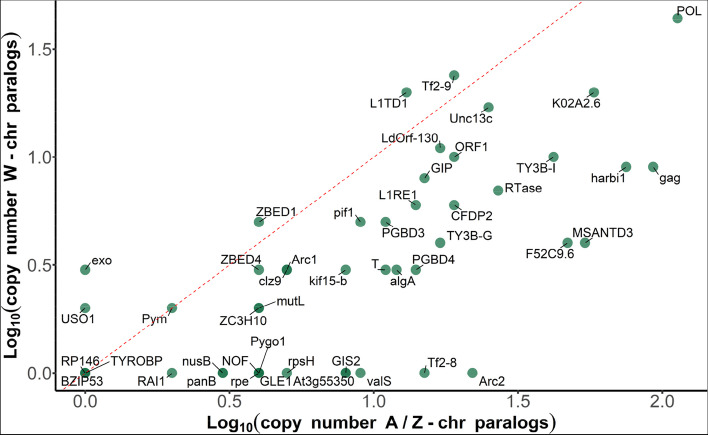
Copy number of W genes and their corresponding autosome/Z chromosome paralogs. The green dots represent the W genes which have paralogs on autosome or Z chromosome; each gene is marked with gene names; some dots overlapped and details are shown in Table S4 in the Additional File 1

Sex-limited chromosomes often contain many copies of some genes [7, 25], and so we examined copy number for genes on the W chromosome and their most similar paralog (Fig. 6, Additional File 1: Table S4). Overall, we observed lower copy number of W genes than that of their autosome/Z chromosome paralogs.

Given the rapid apparent decay of W chromosome paralogs (Fig. 5), we examined the Gene Ontology enrichment for W expressed genes (>0.5 FPKM) (Tables 1 and 2), and observed statistical enrichment of terms related to functions in DNA recombination, the nucleosome, chromatin, and DNA binding. Together, these results suggest that many genes retained with functional expression on the W chromosome are related to mitotic and meiotic processes, much of it within the female gonad.

**Table 1:** Functional enrichment of 66 significantly expressed genes (FPKM > 0.5 from mixed sample) on *Ostrinia furnacalis* W chromosome

PANTHER GO	FDR	Expressed W genes
Biological		
Negative regulation of DNA recombination	7.64 e−11	5
Nucleosome assembly	8.35 e−10	5
Chromosome condensation	2.48 e−8	5
Telomere maintenance	2.67 e−02	2
Molecular		
Nucleosomal DNA binding	1.16 e−11	5
Structural constituent of chromatin	1.13 e−10	5
Double-stranded DNA binding	1.66 e−7	7
Cellular		
Nucleosome	1.68 e−10	5
Chromatin	6.12 e−7	6

**Table 2:** Functional enrichment of 48 significantly expressed genes (FPKM > 0.5 from female gonad) on *Ostrinia furnacalis* W chromosome

PANTHER GO term	FDR	Expressed W genes
Biological		
Negative regulation of DNA recombination	2.04e−10	5
Nucleosome assembly	2.22e−9	2
Chromosome condensation	7.70e-08	5
Molecular		
Nucleosomal DNA binding	3.09e−11	5
Structural constituent of chromatin	3.02e−10	5
Double-stranded DNA binding	8.28e−05	6
Cellular		
Nucleosome	4.46e−10	5
Chromatin	3.19e−6	6

## Discussion

Here we report a high quality, chromosome-level genome for *O. furnacalis*. Our reference genome includes a single, contiguous sequence for the female-specific W chromosome, allowing us to query the content of this unique region of the genome.

### Sequence characteristics of W chromosome

W and Y chromosomes are often enriched for pseudogenes, as the lack of recombination in these regions leads to high rates of gene silencing [11]. At the same time, these regions often accumulate repeat sequences [10, 11, 26], resulting in significantly higher repeat density compared to other chromosomes [12, 26]. Consistent with this, although we found that the number of annotated protein-coding genes of W chromosome is indeed the largest of all the chromosomes in the genome (Fig. 1B), as well as the number of pseudogenes (Fig. 1), and those genes that retain functional coding sequence have low overall transcriptional activity (Fig. 1D) [11, 14]. We also found the W is enriched for repetitive elements in *O. furnacalis* (Additional File 1: Figure S5 and S6), with the number of *Maverick* elements particularly higher on the W chromosome compared to the rest of the genome (Additional File 1: Figure S5).

### Conservation of sex chromosomes across Lepidoptera species

We searched orthologs to Z-linked genes of *O. furnacalis* in other lepidopterans and found that although the Z chromosome shows clear strong conservation [16] (Fig. 2), the *O. furnacalis* W chromosome is only conserved with *C. medinalis*, also a member of Crambidae (Fig. 2B-G). This is consistent with previous work suggesting that the composition of the W varies even among species within the same family [27]. W chromosome evolution can be rapid and consistent with this the W chromosomes between two *Pieris* sister species exhibit substantial divergence [10]. Our data also support the rapid evolution W chromosome. Although *O. furnacalis* has some W–W homologs with *C. medinalis*, the W chromosomes have diverged at a dramatically higher pace than the autosomes and the Z chromosome (Fig. 2C).

### Evolutionary history of the sex chromosomes in *O. furnacalis*

Karyotype work has revealed a complex history of the W chromosome, including the repeated origin of neo-W chromosomes in many lepidopteran lineages. The evolutionary history of the W chromosome in Lepidoptera differs from the canonical model of sex chromosome formation in that it was recruited, possibly from a B element, after the formation of the Z chromosome [16], at the common root of Ditrysia and Tischerioidea [14, 28]. Lineages ancestral to this recruitment exhibit Z0/ZZ karyotypes [16]. The B-chromosome origin of the W is supported by the fact that in some lepidopteran lineages, W chromosome bears no homology to the Z [11, 15, 17].

Some have recently argued that the rapid evolution of the W makes it difficult to differentiate between canonical (Z-homology) and non-canonical (B chromosome) origins of the W chromosome if that origin is deep in the past [10]. The definitive test of these alternatives, direct evidence of B chromosome recruitment to a W chromosome, is difficult to envisage, as it would require both stability of the ancestral B chromosome in an outgroup lineage, unlikely given the instability of B chromosomes in general, as well as widespread homology of the derived W chromosome to the ancestral B. Without this direct evidence, we must rely on indirect evidence from Z-W homology.

We observe little similarity in gene content between the W and Z chromosome (Fig. 4), a steady rate of gene duplication to the W chromosome from throughout the genome (Fig. 5), and reduced intron number for many W paralogs compared to their most similar autosomal/Z-linked copy suggests that this likely often occurs through retrotransposition across the W chromosome. We note that the reduction in introns of W genes compared to their Z homologs is largely consistent with a B chromosome origin of the W. Genes retrotransposed or otherwise duplicated to the W chromosome will immediately experience the full degenerative effects of a non-recombining region [1, 29], and consistent with this, we observe high levels of pseudogenization (Fig. 1) on the W chromosome. However, those genes on the W that retain functional coding sequence and expression (e.g., non-pseudogenized) are enriched for mitotic and meiotic functions and display Gene Ontology term enrichments related to recombination, chromosome packaging, and replication (Tables 1 and 2). It has been suggested that W chromosomes may differ from Y chromosomes in that they do not acquire genes related to female gonadal function [3, 12] in the same way that Y chromosomes retain genes related to male function [5–8]; however, our results suggest that this may not be a generalized pattern of W chromosomes. Indeed, the W chromosome in *O. furnacalis* are enriched for functions related to female meiosis and mitosis, possibly due to TE activity.

## Conclusions

Our study presents a chromosome-level genome from a Lepidipteran, *O. furnacalis*. Comparative analysis reveals the deep conservation of the Z chromosome across Lepidoptera, but little conservation of W chromosome in related species or with the Z chromosome, which supports the non-canonical origin of the W chromosome. *O. furnacalis* W chromosome has accumulated significant repetitive elements and experienced rapid gene gain from the remainder of the genome, with most genes exhibiting pseudogenization after duplication to the W. The genes that retain significant expression are largely enriched for functions in DNA recombination, the nucleosome, chromatin, and DNA binding, likely related to meiotic and mitotic processes within the female gonad.

## Methods

### Samples

*O. furnacalis* larvae were collected from a corn field at Beijing, China, in July 2020, and fed with an artificial diet in the laboratory of China Agricultural University. The incubator environment was set at 26 ± 1℃ and 50 ± 5% relative humidity on a photoperiod (light: dark = 16:8).

### Genome sequencing and assembly

Genomic DNA was extracted from a female laboratory-fed pupa using the sodium dodecyl sulfate (SDS) extraction method [30]. The SMRTbell library was constructed for sequencing using the SMRTbell Express Template Prep kit 2.0 (Pacific Biosciences). From this single individual, we obtained 32.96 Gb circular consensus sequencing (CCS) data from one cell of the PacBio Sequel II platform at Biomarker Co., Ltd., Qingdao, China (Additional File 1: Table S5). High accuracy PacBio Hifi data were assembled using hifiasm (v0.12) software to obtain a draft genome [31]. We used purge_haplotigs [32] to remove redundant contigs and generate a non-redundant assembled genome.

### Hi-C scaffolding

We constructed Hi-C libraries (300–700 bp insert size) using one female pupa, following Rao et al. [33], and sequenced them with pair-end 150 Illumina Hiseq at Biomarker Co., Ltd., Qingdao, China; 53.05 Gb of clean data were produced after filtering adapter sequences, primer sequences, and low-quality data (Additional File 1: Table S6). The resulting trimmed reads were aligned to the draft assembly with BWA (v0.7.10-r789), retaining only uniquely aligned read pairs with mapping quality >20 for further analysis. Invalid read pairs, including dangling-end, self-cycle, re-ligation, and dumped products, were removed by HiC-Pro (v2.8.1) [34].

The 56.25% of unique mapped read pairs represent valid interaction pairs and were used for correction, clustering, ordering, and orientation of scaffolds into chromosomes with LACHESIS [35]. Before chromosome assembly, we performed a preassembly for error correction of contigs which required the splitting of contigs into segments of 50 kb on average. Then Hi-C data were mapped to these segments using BWA (v0.7.10-r789). The uniquely mapped data were retained for the assembly, and any two segments which showed inconsistent connection with information from the raw scaffolds were checked manually. Parameters for running LACHESIS included: CLUSTER_MIN_RE_SITES = 27; CLUSTER_MAX_LINK_DENSITY = 2; ORDER_MIN_N_RES_IN_TRUNK = 15; ORDER_MIN_N_RES_IN_SHREDS = 15. After this step, placement and orientation errors exhibiting obvious discrete chromatin interaction patterns were manually adjusted. Finally, we constructed a heatmap based on the interaction signals of valid mapped read pairs between chromosomes.

### Genome annotation

Transposable elements (TEs) were identified by a combination of homology-based and de novo approaches. We first carried out a de novo repeat prediction using RepeatModeler2 (v2.0.1) [36], which implements RECON (v1.0.8) [37] and RepeatScout (v1.0.6) [38]. Then the predicted results were classified using RepeatClassifier [36] by means of repbase (v19.06) [39], REXdb (v3.0) [40], and Dfam (v3.2) [41]. Secondly, we performed a de novo prediction for long terminal repeats (LTRs) using LTR_retriever (v2.8) [42] via LTRharvest (v1.5.9) [43] and LTR_finder (v2.8) [44]. A non-redundant species-specific TE library was constructed by combining the de novo TE library above with the known databases. Final TE sequences in the *O. furnacalis* genome were identified and classified by homology search against the library using RepeatMasker (v4.10) [45]. Tandem repeats were annotated by Tandem Repeats Finder (TRF v409) [46] and MIcroSAtellite identification tool (MISA v2.1) [47].

We integrated de novo prediction, homology search, and transcript-based assembly to annotate protein-coding genes. The de novo gene models were predicted using Augustus (v2.4) [48] and SNAP (2006-07-28) [49], both* ab initio* gene-prediction approaches. For the homolog-based approach, we used GeMoMa (v1.7) [50] with reference gene models from *Drosophila melanogaster*, *B. mori*, *Chilo suppressalis*, and *Galleria mellonella*. For the transcript-based prediction, total RNA was extracted from a mixture sample containing egg, larva, pupa, and adult whole body of females and males using TRIZOL reagent (Invitrogen) and sent to BioMarker for cDNA library generation and sequencing on an Illumina NovaSeq 6000 platform (25.82-fold coverage of the genome, Additional File 1: Table S7). RNA-sequencing data were mapped to the reference genome using Hisat (v2.0.4) [51] and assembled with Stringtie (v1.2.3) [52]. GeneMarkS-T (v5.1) [53] was used to predict genes based on the assembled transcripts. PASA (v2.0.2) [54] was used to predict genes based on the unigenes assembled by Trinity (v2.11) [55]. Gene models from these different approaches were combined with EVM (v1.1.1) [56] and updated by PASA (v2.0.2) [54]. The final gene models were annotated by searching the GenBank Non-Redundant (NR, 20200921), TrEMBL (202005), Pfam (v33.1) [57], SwissProt (202005) [58], eukaryotic orthologous groups (KOG, 20110125), gene ontology (GO, 20200615), and Kyoto Encyclopedia of Genes and Genomes (KEGG, 20191220) databases.

We used tRNAscan-SE (v1.3.1) [59] with default parameters to identify transfer RNAs (tRNAs) and barrnap (v0.9) with default parameters to identify the ribosomal RNAs (rRNAs) based on Rfam (v12.0) [60]. miRNAs were identified with miRbase [61]. Small nucleolar RNA (snoRNAs) and small nuclear RNA (snRNAs) were identified with Infernal (v1.1.1) [62], using the Rfam (v12.0) database [60].

Pseudogenes have similar sequences to functional genes, but may have lost their biological function because of mutations. GenBlastA (v1.0.4) [63] was used to scan the whole genome after masking predicted functional genes. Putative candidates were then analyzed by searching for premature stop codon and frameshift mutations using GeneWise (v2.4.1) [64].

### Comparative genomics and phylogenetic reconstruction

Protein sequence alignments between *O. furnacalis* and six other lepidopteran species (*B. mori*, *C. medinalis, C. pomonella*, *S. exigua, S. fugiperda,* and *T. ni*) (Additional File 1: Table S8) were performed with diamond (-e < 1e-5), then alignment results were analyzed and the homologous chromosomal regions were identified with MCScanX (MATCH_SCORE: 50, MATCH_SIZE: 5, GAP_PENALTY: -1, OVERLAP_WINDOW: 5, E_VALUE: 1e-05, MAX GAPS: 15). The synteny relationships among chromosomes were displayed using circos (v0.69–9).

We used the protein sequences of *O. furnacalis* and eleven other insect species (*B. mori, C. suppressalis, C. medinalis, S. frugiperda, S. litura, Danaus plexippus, Melitaea cinxia, Papilio xuthus, C. pomonella*, *Plutella xylostella,* and *D. melanogaster*) for phylogenetic analysis (Additional File 1: Table S8), keeping only the longest transcript of each gene for analysis, and using OrthoFinder (v2.5.4) [65] with default settings to identify orthologues and homologs. To infer the phylogeny of these insects, multiple sequence alignments of single-copy orthologs were performed using MAFFT (v7.471) [66]. Then we extracted conserved sequences with gblocks (v0.91b) [67] and concatenated them to a single sequence alignment. The resulting sequence alignment was used to construct a maximum likelihood phylogenetic tree using IQ-TREE (v1.6.12) [68] (outgroup: *D. melanogaster*). Divergence times between various species were estimated by MCMCtree in PAML (v4.9j) [69]. Three standard divergence time points from the TimeTree database (http://timetree.org/) were used for calibration: (*O. furnacalis*, *C. medinalis*)—*C. suppressalis*, 66.2–71.4 million years ago (Mya), *S. frugiperda*—S. *litura*, 63.4–122.1 million years ago (Mya), and *D. plexippus*—*M. cinxia*, 69.4–111.5 Mya. The tree was visualized using FigTree (v1.4) (http://tree.bio.ed.ac.uk/software/figtree/). The gene count table from OrthoFinder (v2.5.4) was used as inputs to examine the expansion and contraction of each gene family in cafe (v5.1.0) [70].

### Detection of sex chromosomes

In order to identify the sex chromosomes, we performed genome resequencing with five female and male pupae. High-quality clean data (24.95~28.80-fold coverage of the genome) were obtained through the pair-end 150 Illumina Hiseq platform at Biomarker Co., Ltd., Qingdao, China (Additional File 1: Table S7). The clean data were aligned to reference genome with Bwa-men (v0.7.17). We compared the coverage differences between male and female samples [71] to distinguish the Z, W, and autosomes. Specifically, we used the genomecov and groupby in Bedtools (v2.30.0) to obtain a per-base median coverage depth for each chromosome and normalized them by the mean of all chromosome median coverages for each sample. Normalized coverage depth was averaged by sex to produce a coverage depth per chromosome for each sex. Then we compared coverage depth between sexes for each chromosome and calculated log_2_ male:female (M:F) coverage ratio [log_2_(M:F coverage)]. Autosomes have an equal coverage between sexes [log_2_ (M:F coverage) = 0], while the Z chromosome should show approximately twofold greater coverage in the males [log_2_(M:F coverage) = 1]. The W chromosome should show a strong female-biased coverage pattern. We also calculated the M:F median coverage ratio along each chromosome with nonoverlapping 1000 bp windows.

### W-gene homologs search and calculation of synonymous divergence (dS)

Predicted protein sequences were used to detect reciprocal best hits between the W chromosome and the remainder of the genome using getRBH.pl (https://github.com/Computational-conSequences/SequenceTools) for *O. furnacalis*. For each pair of orthologous genes, we deleted stop codons and aligned the coding sequences using macse (v2.05) [72], then extracted conserved sequences with gblocks (v0.91b) [67]. The resulting alignments were used as inputs of CODEML in PAML (v4.9j) [69] to estimate pairwise synonymous divergence with settings runmode = -2, seqtype = 1 and CodonFreq = 2. Since divergence estimates are not reliable for saturated sites, we excluded orthologs with dS > 3 [73].

The homolog search of *O. furnacalis* W in *C. pomonella*, *C. medinalis*, *S. exigua*, *S. fugiperda*, and *T.ni* genome also used getRBH.pl. We assessed enrichment of Gene Ontology [74, 75] terms for W gene content.

### W-gene expression level

We used two different RNA-Seq datasets to determine W expression, the mixture sample of RNA-Seq data from eggs, larvae, pupae, and adults used for genome annotation (described above), and data from female gonad, as sex-limited chromosomes are often largely expressed in the gonad. For female gonad, RNA from five gonad samples of adult females was sequenced on an Illumina NovaSeq 6000 platform (10.14-fold coverage of the genome, Additional File 1: Table S7). For each dataset separately, we used fastp (v0.20.0) [76] to filter out low-quality reads and to remove adapters with the default parameters. Then we mapped the clean reads to the *O. furnacalis* reference genome using HISAT2 (v2.1.0) [51]. The FPKM (fragments per kilobase of exon per million fragments mapped) of each gene was determined using Stringtie (v2.1.4) [52] based on the annotated GFF file. For female gonad, we used averaged FPKM of five samples.

### Copy number variation of W and Z/autosome genes

The amino acid sequences of protein-coding genes from whole genome were used as the input of the blastp mapping against the Swiss-Prot database to assign gene symbols (abbreviations for the gene names). We BLASTed each W gene to the remainder of the genome to identify the best hit and only retained orthologous genes on the W chromosome and Z/autosomes that consistently mapped to the same protein (reciprocal best BLAST hit) and calculated their copy number following the protocol in Mueller et al. [77] .

### Supplementary Information

**Additional file 1:** **Figure S1.** Genome survey of *Ostrinia furnacalis* using k-mer analysis. **Figure S2.** The genome-wide Hi-C interaction maps of 32 chromosomes in *Ostrinia furnacalis*. The map indicates that intrachromosome interactions were strong while interchromosome interactions were weak. The shading gradient represents the chromosome interactions. **Figure S3.** Synteny analysis between *Ostrinia furnacalis* and *Spodoptera litura* chromosomes. Chromosomes of *Ostrinia furnacalis *are shown in the left, number 3 represent the W chr and 1 represents the Z chr. The chromosomes of *Spodoptera litura* are shown in the right. **Figure S4.** Annotation and evaluation of protein-coding genes. a. Genes annotated via *ab* *initio*, homology-based and RNA-seq methods. b-e. Comparison of *Ostrinia furnacalis* gene features with other lepidopteran genomes. **Figure S5.** The number of repeat sequences in W chromosome (LG3). **Figure S6.** The number (a-b), density (c-d) and proportion (e-f) of repeat sequence in all chromosomes (LG1-LG32). **Table S1.** Chromosome-level assembled Lepidoptera genomes. **Table S2.** Assessments of assembled genome. **Table S3.** Genomic annotation of *Ostrinia furnacalis*. **Table S4.** Copy number for W and autosomal/Z chromosome paralogs. **Table S5. **Statistics of genomic sequencing data of *Ostrinia furnacalis* by PacBio Sequel II. **Table S6.** Statistics of genomic sequencing data of *Ostrinia furnacalis* by Hi-C. **Table S7.** Statistics of genomic resequencing data of female and male pupae and transcriptome sequencing of female gonads and a mixed sample. **Table S8.** The download address of insect species protein sequences used for comparative genomics analysis and phylogenetic reconstruction.

## Data Availability

All raw sequencing data used in this study have been deposited in the Genome Sequence Archive (GSA) database (https://ngdc.cncb.ac.cn/gsa/) under accession number CRA008610 [78]. The final genome assembly, annotations, and other data have been deposited in figshare (https://figshare.com/projects/Ostrinia_furnacalis_assembly_and_annotation/129992) [79, 80, 82,83,81]. The final genome assembly was also submitted to the NCBI genome resource with accession JBBYJR000000000 [84].

## Abbreviations

Hi-C: High-through chromosome conformation capture
CCS: Circular consensus sequencing
BUSCO: Benchmark of Universal Single-Copy Orthologs
tRNA: TransferRNAs
rRNA: Ribosomal RNAs
miRNA: MicroRNAs
snoRNAs: Small nucleolar RNA
snRNAs: Small nuclear RNA
TEs: Transposable elements
KOG: Eukaryotic orthologous group
GO: Gene ontology
KEGG: Kyoto Encyclopedia of Genes and Genomes
