# Supplementary material for: Gene gain and loss from the Asian corn borer W chromosome
Source: BMC Biol. 2024 May 1;22:102. doi: 10.1186/s12915-024-01902-4 (PMC11064298; doi:10.1186/s12915-024-01902-4)
Supplement: Supplementary file 1 — Additional file 1: Figure S1. Genome survey of Ostrinia furnacalis using k-mer analysis. Figure S2. The genome-wide Hi-C interaction maps of 32 chromosomes in Ostrinia furnacalis. The map indicates that intrachromosome interactions were strong while interchromosome interactions were weak. The shading gradient represents the chromosome interactions. Figure S3. Synteny analysis between Ostrinia furnacalis and Spodoptera litura chromosomes. Chromosomes of Ostrinia furnacalis are shown in the left, number 3 represent the W chr and 1 represents the Z chr. The chromosomes of Spodoptera litura are shown in the right. Figure S4. Annotation and evaluation of protein-coding genes. a. Genes annotated via ab initio, homology-based and RNA-seq methods. b-e. Comparison of Ostrinia furnacalis gene features with other lepidopteran genomes. Figure S5. The number of repeat sequences in W chromosome (LG3). Figure S6. The number (a-b), density (c-d) and proportion (e-f) of repeat sequence in all chromosomes (LG1-LG32). Table S1. Chromosome-level assembled Lepidoptera genomes. Table S2. Assessments of assembled genome. Table S3. Genomic annotation of Ostrinia furnacalis. Table S4. Copy number for W and autosomal/Z chromosome paralogs. Table S5. Statistics of genomic sequencing data of Ostrinia furnacalis by PacBio Sequel II. Table S6. Statistics of genomic sequencing data of Ostrinia furnacalis by Hi-C. Table S7. Statistics of genomic resequencing data of female and male pupae and transcriptome sequencing of female gonads and a mixed sample. Table S8. The download address of insect species protein sequences used for comparative genomics analysis and phylogenetic reconstruction. [file 12915_2024_1902_MOESM1_ESM.docx]

**Additional File 1:**


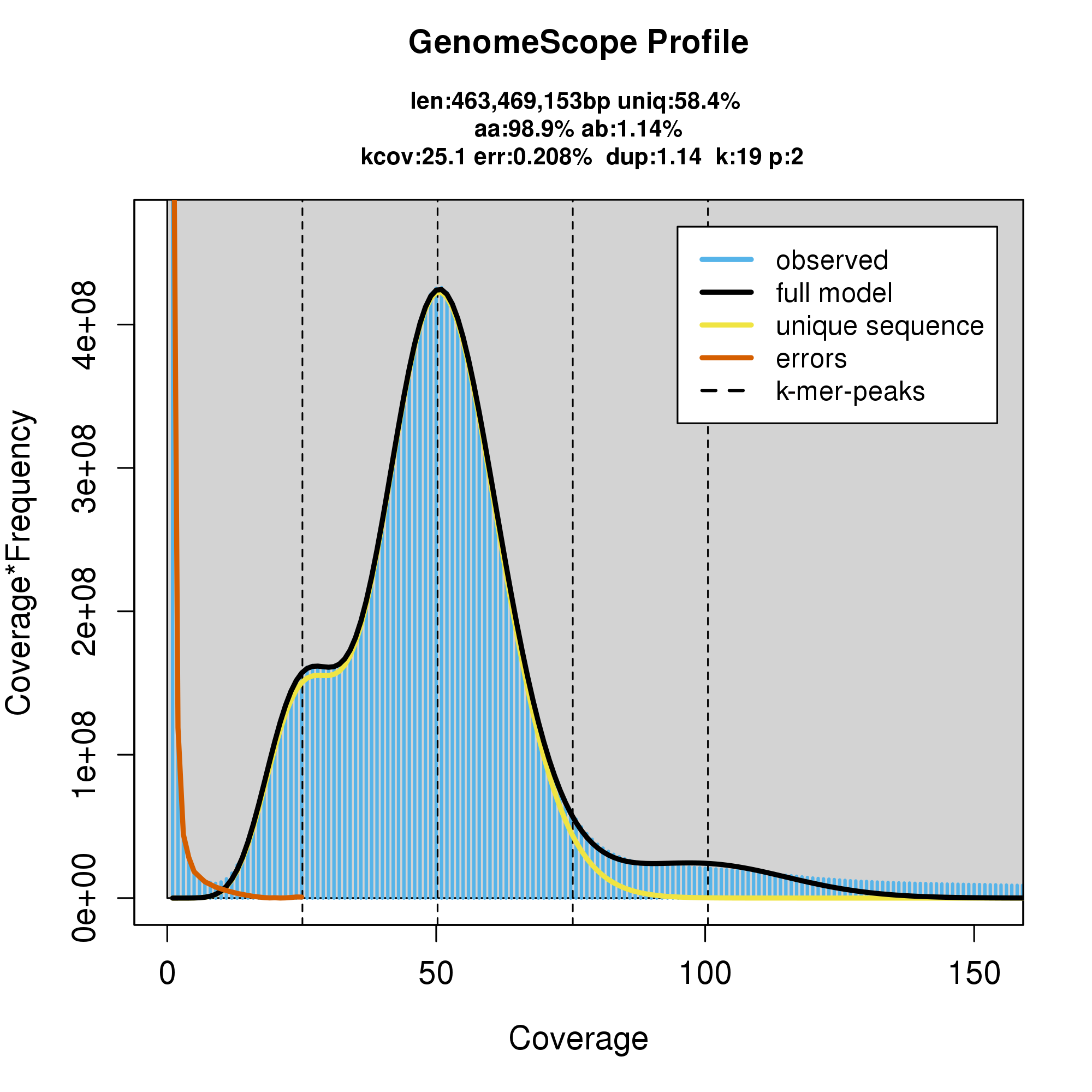


**Figure S1. Genome survey of *Ostrinia furnacalis* using k-mer analysis.**


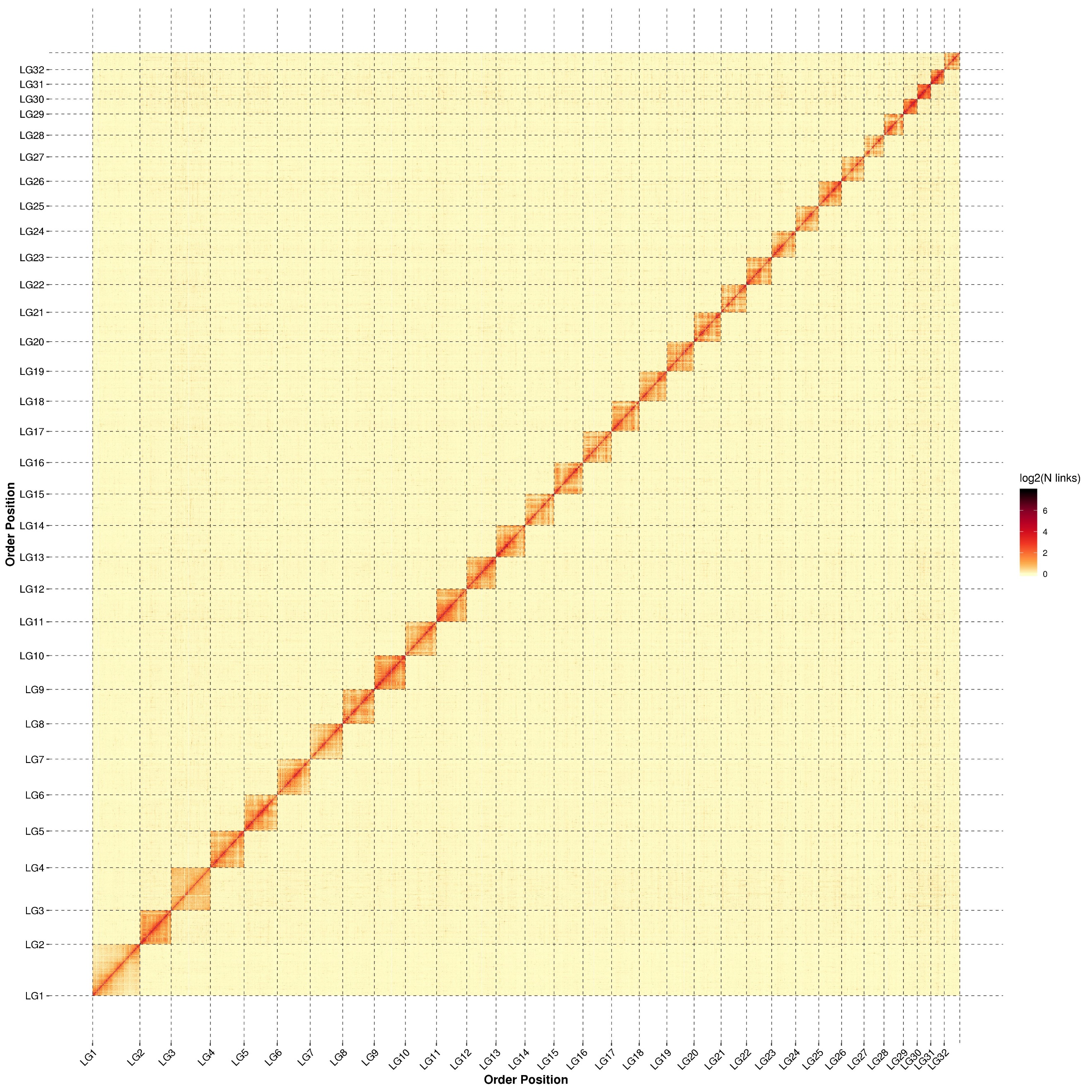


**Figure S2. The genome-wide Hi-C interaction maps of 32 chromosomes in *Ostrinia furnacalis*.** The map indicates that intrachromosome interactions were strong while interchromosome interactions were weak. The shading gradient represents the chromosome interactions.


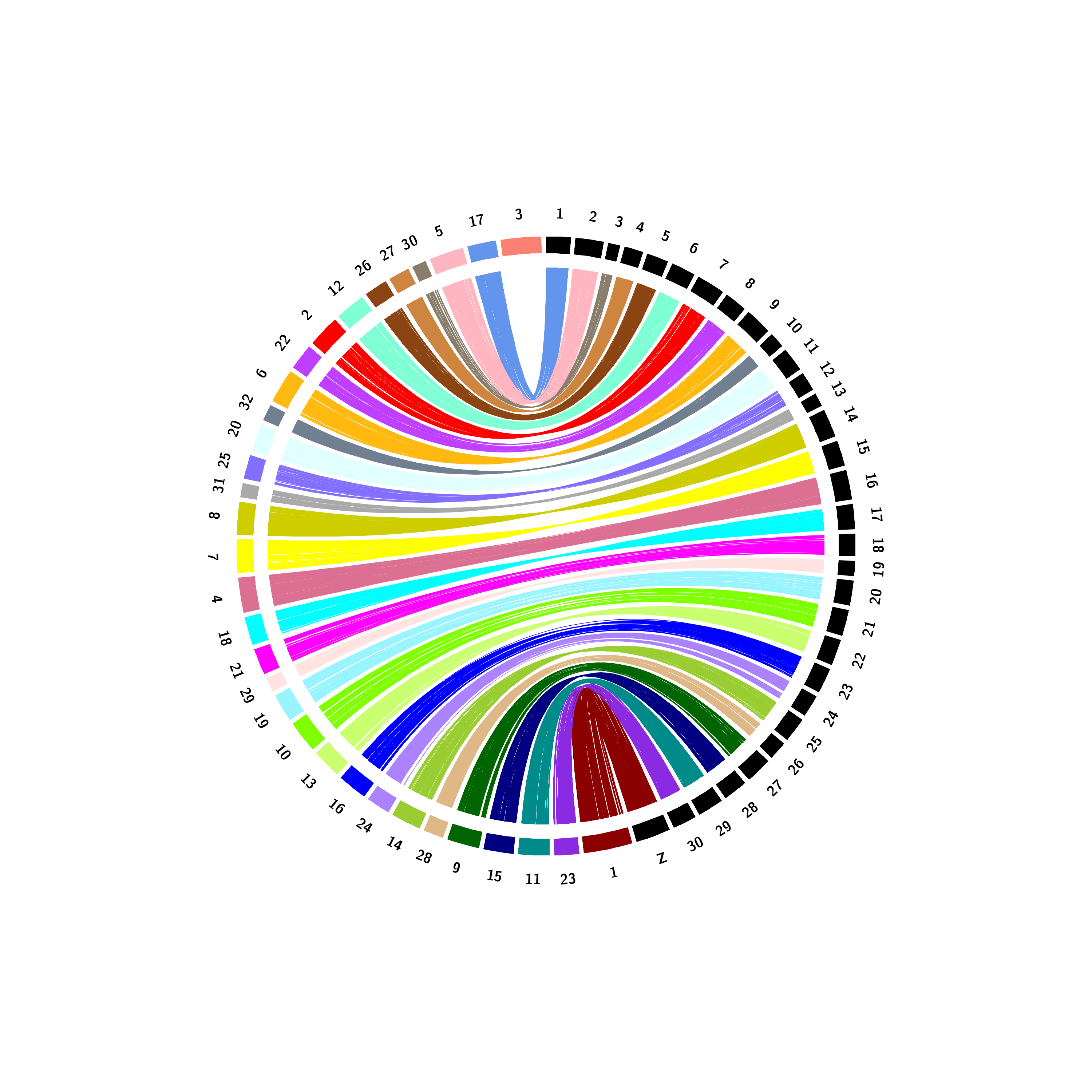


***O. furnacalis***

***S. litura***

**Figure S3. Synteny analysis between *Ostrinia furnacalis* and** ***Spodoptera litura* chromosomes. Chromosomes of *Ostrinia furnacalis* are shown in the left, number 3 represent the W chr and 1 represents the Z chr. The chromosomes of *Spodoptera litura* are shown in the right.**


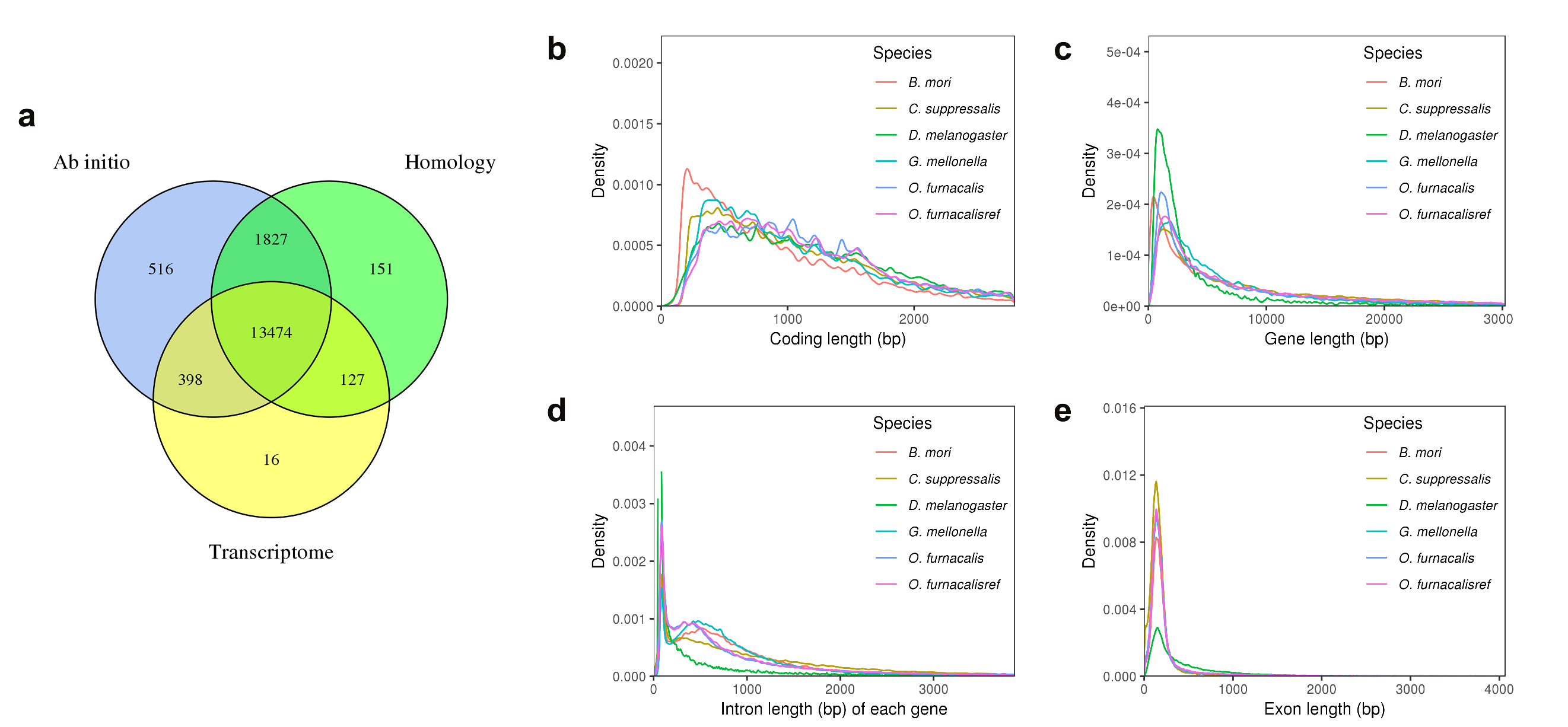


**Figure S4. Annotation and evaluation of protein-coding genes. a.** Genes annotated via *ab initio*, homology-based and RNA-seq methods. **b-e.** Comparison of *Ostrinia furnacalis* gene features with other lepidopteran genomes.


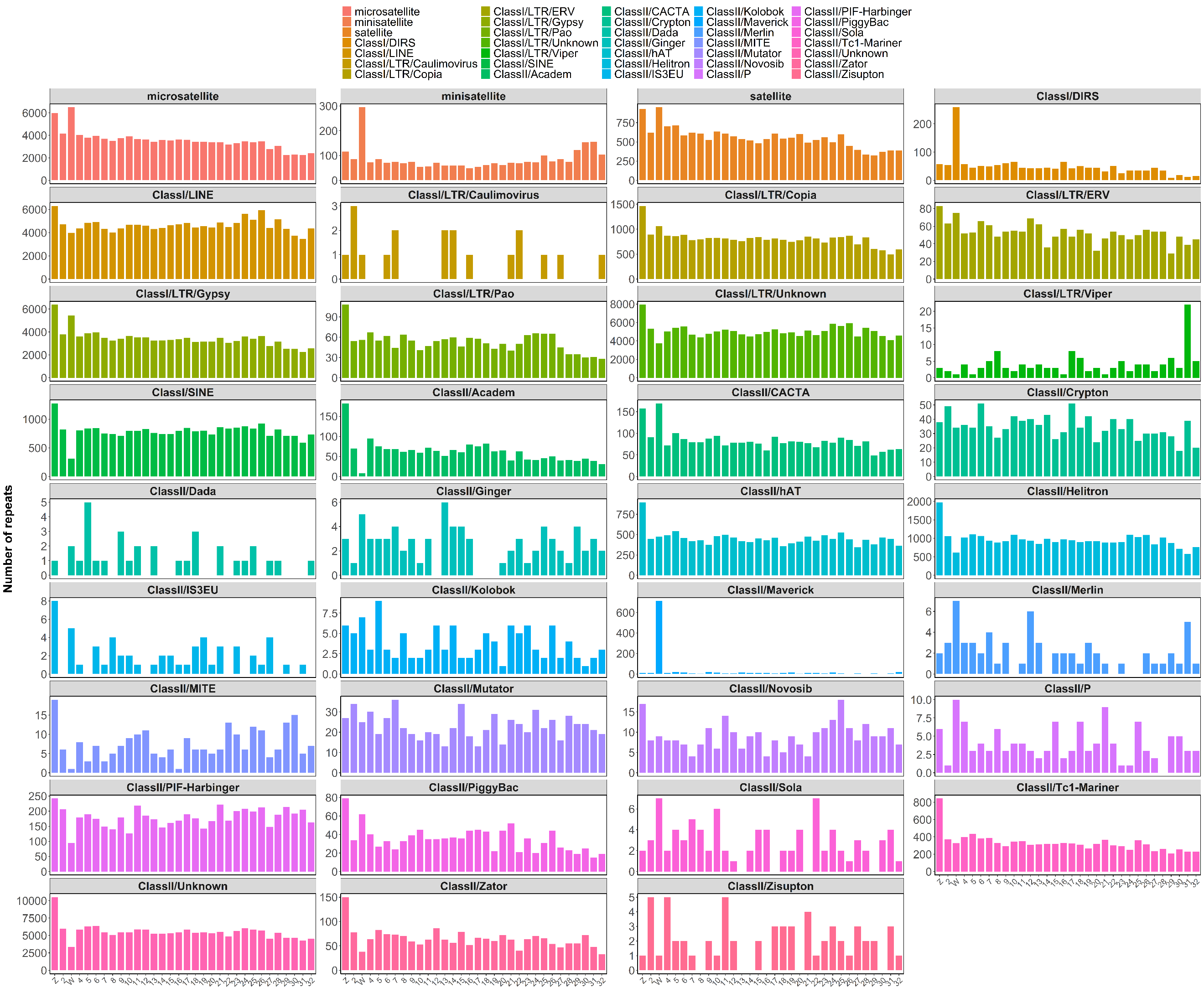


**Figure S5. The number of repeat sequences in W chromosome (LG3).**

**
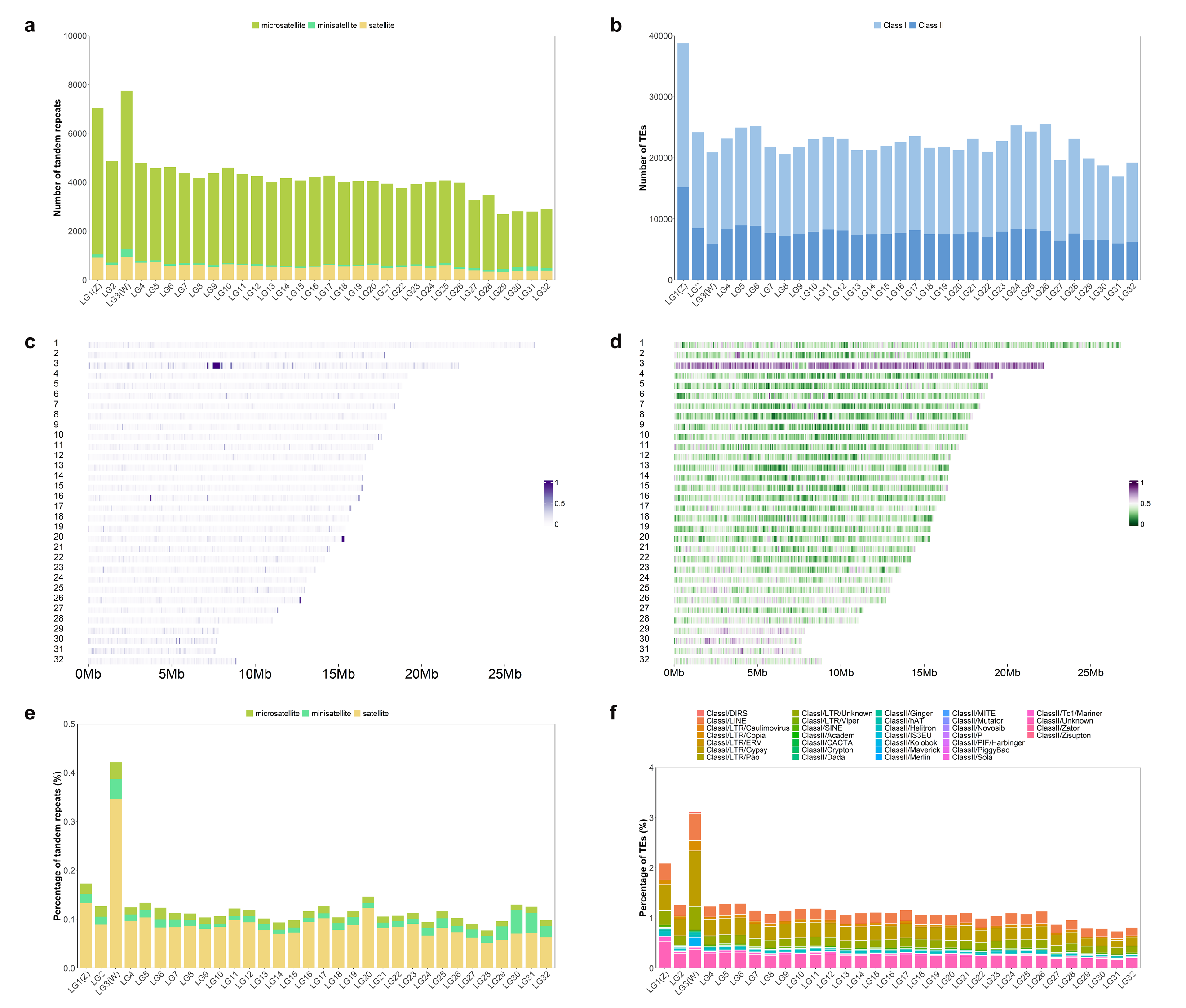
**

**Figure S6. The number (a-b), density (c-d) and proportion (e-f) of repeat sequence in all chromosome (LG1-LG32).**

**Table S1 Chromosome-level assembled Lepidoptera genomes**

|  | *O. furnacalis* | *C. medinalis* | *C. suppressalis* | *C. pomonella* | *T. ni* | *H. melpomene* | *B. mori* |
| --- | --- | --- | --- | --- | --- | --- | --- |
| Genome size (Mb) | 493.1 | 528.3 | 824.4 | 772.9 | 368.2 | 269.0 | 460.3 |
| Karyotype | 2n=62 | 2n=60 | 2n=58 | 2n=56 | 2n=54 | 2n=42 | 2n=56 |
| Number of contigs | 57 | 4671 | – | 2221 | 26605 | – | - |
| Number of scaffolds | 43 | 3248 | 27144 | 1717 | 6181 | 3807 | 696 |
| Number of assembled chromosomes | 30A+Z+W | 29A+Z+W | 28A + Z | 27A+Z+W | 26A+Z+W | 20A+Z | 27A+Z |
|  |  |  |  |  |  |  |  |
| **Genome assembly quality** | | | | | | | |
| Contig N50 (Mb) | 15.7 | 0.5 | 0.3 | 0.9 | 0.6 | 0.1 | 12.2 |
| Scaffold N50 (Mb) | 16.5 | 16.1 | 1.8 | 8.9 | 14.2 | 0.3 | 16.8 |
| Percentage of scaffolds in chromosomes (%) | 86.8 | 89.1 | 92.5 | 97.5 | 90.6 | 82.7 | 87.3 |
| BUSCO genes (%) | 97.7 | 96.4 | 97.5 | 98.5 | 97.8 | 97.4 | 97.7 |
|  |  |  |  |  |  |  |  |
| **Genomic features** | | | |  |  |  |  |
| Repeat (%) | 41.5 | 39.5 | 46.4 | 42.9 | 20.5 | 24.9 | 46.8 |
| G+C (%) | 37.6 | 38.5 | 36.9 | 37.4 | 35.6 | – | 38.2 |
|  |  |  |  |  |  |  |  |
| **Gene annotation** | | | | | | | |
| Number of genes | 16509 | 15045 | 15653 | 17184 | 14043 | 12669 | 14623 |

**Table S2. Assessments of assembled genome**

| **BUSCOs** | | | | | | | | | |  |
| --- | --- | --- | --- | --- | --- | --- | --- | --- | --- | --- |
| Complete BUSCOs(C) | Complete and single-copy BUSCOs(S) | | Complete and duplicated BUSCOs(D) | | | Fragmented BUSCOs(F) | Missing BUSCOs(M) | | Total Lineage BUSCOs |  |
| 5218(98.7%) | 5205(98.5%) | | 13(0.2%) | | | 19(0.4%) | 49(0.9%) | | 5286 |  |
| **DNA-seq data mapping** | | | | | | | | | | |
| Total_reads | Mapped reads | | | Mapped (%) | Properly mapped | | | Properly mapped (%) | | |
| 183,973,226 | 182,267,035 | 99.07% | | | 175,334,598 | | | 95.30% | | |

**Table S3. Genomic annotation of *Ostrinia furnacalis*.**

|  | Annotated Number | Annotated Ratio |
| --- | --- | --- |
| tRNA | 7,710 | – |
| rRNA | 73 | – |
| miRNA | 39 | – |
| pseudogenes | 167 | – |
| Annotation of protein-coding genes | | |
| GO_Annotation | 12013 | 72.77 |
| KEGG_Annotation | 12033 | 72.89 |
| KOG_Annotation | 9314 | 56.42 |
| Swissprot_Annotation | 10864 | 65.81 |
| TrEMBL_Annotation | 15901 | 96.32 |
| eggNOG_Annotation | 11746 | 71.15 |
| nr_Annotation | 16201 | 98.13 |
| All_Annotated | 16213 | 98.21 |

**Table S4. Copy number for W and autosomal/Z chromosome paralogs.**

|  | **Aurosomes & Z** | | **W chr** |
| --- | --- | --- | --- |
| Gene name | Copy number | Chromosomes | Copy number |
| *algA* | 12 | LG9/LG11/LG13/LG19/LG22/LG24/LG27/LG29/LG31/LG32 | 3 |
| *Arc1* | 5 | LG16/LG21/LG25/LG26/LG28 | 3 |
| *Arc2* | 22 | LG1/LG5/LG6/LG11/LG12/LG14/LG15/LG18/LG19/LG20/LG21/LG23/LG26 | 1 |
| *At3g55350* | 8 | LG8/LG11/LG18/LG21/LG22/LG25 | 1 |
| *BZIP53* | 1 | LG19 | 1 |
| *CFDP2* | 19 | LG2/LG6/LG7/LG8/LG10/LG11/LG13/LG14/LG16/LG19/LG20/LG22/LG25/LG26/LG30/LG32 | 6 |
| *clz9* | 5 | LG1/LG8/LG11/LG26/LG32 | 3 |
| *exo* | 1 | LG21 | 3 |
| *F52C9.6* | 47 | LG2/LG4/LG5/LG6/LG7/LG8/LG8/LG9/LG10/LG11/LG12/LG13/LG14/LG15 | 4 |
| *gag* | 93 | LG1/LG2/LG4/LG5/LG6/LG7/LG8 | 9 |
| *GIP* | 15 | LG2/LG4/LG9/LG10/LG14/LG15/LG18/LG19/LG25/LG26/LG27 | 8 |
| *GIS2* | 8 | LG9/LG10/LG11/LG21/LG24/LG27/LG28 | 1 |
| *GLE1* | 4 | LG7/LG18/LG20/LG22 | 1 |
| *harbi1* | 75 | LG1/LG2/LG4/LG6/LG7/LG9/LG10/LG11/LG12/LG13 | 9 |
| *K02A2.6* | 58 | LG1/LG4/LG5/LG6/LG8/LG9/LG9/LG10/LG11/LG12/LG13/LG14/LG15 | 20 |
| *kif15-b* | 8 | LG4/LG14/LG19/LG20/LG22/LG23/LG28/LG32 | 3 |
| *L1RE1* | 14 | LG2/LG8/LG9/LG9/LG10/LG13/LG14/LG15/LG19/LG25/LG26/LG28 | 6 |
| *L1TD1* | 13 | LG2/LG5/LG6/LG16/LG17/LG18//LG19/LG24/LG28/LG32 | 20 |
| *LdOrf-130* | 17 | LG9/LG11/LG13/LG14/LG16/LG18/LG20/LG21/LG22/LG26/LG27/LG28 | 11 |
| *MSANTD3* | 54 | LG1/LG2/LG4/LG6/LG7/LG8/LG9/LG10/LG11/LG13/LG14/LG15 | 4 |
| *mutL* | 4 | LG7/LG15/LG17 | 2 |
| *NOF* | 4 | LG2/LG8/LG12/LG13 | 1 |
| *nusB* | 3 | LG11/LG14 | 1 |
| *ORF1* | 19 | LG1/LG2/LG4/LG6/LG10/LG11/LG12/LG13/LG14/LG17/LG19/LG20/LG23/LG24/LG27 | 10 |
| *panB* | 3 | LG16/LG24/LG25 | 1 |
| *PGBD3* | 11 | LG1/LG4/LG6/LG8/LG11/LG12/LG12/LG13/LG21/LG30 | 5 |
| *PGBD4* | 14 | LG1/LG4/LG10/LG12/LG13/LG16/LG20/LG21/LG22/LG23/LG25 | 3 |
| *pif1* | 9 | LG1/LG9/LG10/LG17/LG18/LG23/LG24/LG31 | 5 |
| *POL* | 113 | LG1/LG2/LG4/LG5/LG6/LG7/LG8 | 44 |
| *Pygo1* | 4 | LG4/LG5/LG22/LG25 | 1 |
| *Pym* | 2 | LG12/LG15 | 2 |
| *RAI1* | 2 | LG14/LG22 | 1 |
| *RP146* | 1 | LG25 | 1 |
| *rpe* | 4 | LG10/LG14/LG16/LG20 | 1 |
| *rpsH* | 5 | LG2/LG9/LG18/LG31 | 1 |
| *RTase* | 27 | LG2/LG5/LG6/LG8/LG9/LG10/LG11/LG12/LG14/LG16/LG17/LG18/LG19/LG21/LG22/LG24/LG28/LG29/LG31 | 7 |
| *T* | 11 | LG1/LG2/LG7/LG8/LG18/LG21/LG31 | 3 |
| *Tf2-8* | 15 | LG1/LG4/LG5/LG6/LG11/LG15/LG17/LG20/LG21/LG22/LG23/LG24/LG27 | 1 |
| *Tf2-9* | 19 | LG4/LG5/LG8/LG9/LG10/LG12/LG14/LG15/LG16/LG20/LG21/LG22/LG26/LG28/LG29/LG30/LG31 | 24 |
| *TY3B-G* | 17 | LG1/LG8/LG9/LG10/LG11/LG14/LG16/LG17/LG20/LG23/LG23/LG24/LG25/LG26 | 4 |
| *TY3B-I* | 42 | LG2/LG4/LG6/LG7/LG8/LG9/LG10/LG13/LG15/LG16/LG17/LG18 | 10 |
| *TYROBP* | 1 | LG11 | 1 |
| *Unc13c* | 25 | LG1/LG5/LG6/LG8/LG11/LG14/LG15/LG16/LG17/LG18/LG19/LG21/LG22/LG24/LG26/LG30 | 17 |
| *USO1* | 1 | LG18 | 2 |
| *valS* | 9 | LG4/LG8/LG10/LG16/LG17/LG19/LG20/LG22/LG29 | 1 |
| *ZBED1* | 4 | LG6/LG13/LG22/LG25 | 5 |
| *ZBED4* | 4 | LG21/LG25/LG31 | 3 |
| *ZC3H10* | 4 | LG1/LG11/LG26 | 2 |

**Table S5. Statistics of genomic sequencing data of *Ostrinia furnacalis* by PacBio Sequel II**

| Sequence Type | Reads Num | Total Bases (bp) | Reads N50 (bp) | Mean Length (bp) | Longest Read (bp) |
| --- | --- | --- | --- | --- | --- |
| Subreads | 43,968,759 | 515,146,437,892 | 12,514 | 11,716 | 509,876 |
| CCS | 2,535,838 | 32,962,737,921 | 13,153 | 12,999 | 50,267 |

**Table S6. Statistics of genomic sequencing data of *Ostrinia furnacalis* by Hi-C**

| Clean data by Hi-C | | | | | |
| --- | --- | --- | --- | --- | --- |
| ReadSum | BaseSum | GC(%) | N^a^(%) | Q20^b^(%) | Q30^c^(%) |
| 177382279 | 53050949420 | 37.4 | 0 | 97.55 | 93.16 |
| Mapping rate of Hi-C clean data | | | | | |
| Total Read Pairs | Mapped Reads | | Unique Mapped Read Pairs | | |
| 177,382,279 | 276,188,309 | | 118,446,847 | | |

a: The ratio of N in the bases

b: The percentage of bases with Phred quality score ≥ 20

c: The percentage of bases with Phred quality score ≥ 30

**Table S7. Statistics of genomic resequencing data of female and male pupae and transcriptome sequencing of female gonads and a mixed sample**

| Sample | ReadSum (Mb) | BaseSum (Gb) | Q30(%) |
| --- | --- | --- | --- |
| **Genomic resequencing** |  |  |  |
| female1 | 82.2 | 12.3 | 93 |
| female2 | 94.0 | 14.1 | 94 |
| female3 | 91.4 | 13.7 | 94 |
| female4 | 92.3 | 13.8 | 93 |
| female5 | 93.0 | 13.9 | 93 |
| male1 | 93.8 | 14.0 | 93 |
| male2 | 84.4 | 12.6 | 93 |
| male3 | 95.0 | 14.2 | 93 |
| male4 | 93.7 | 14.0 | 92 |
| male5 | 87.7 | 13.1 | 93 |
| **Transcriptome sequencing** |  |  |  |
| female_gonad1 | 21.5 | 6.4 | 94 |
| female_gonad2 | 21.2 | 6.3 | 95 |
| female_gonad3 | 20.1 | 6.0 | 95 |
| female_gonad4 | 18.1 | 5.4 | 95 |
| female_gonad5 | 19.8 | 5.9 | 96 |
| mixed sample | 42.7 | 12.7 | 95 |

**Table S8. The download address of insect species protein sequences used for comparative genomics analysis and phylogenetic reconstruction**

| **Species** | **Download** |
| --- | --- |
| *Bombyx mori* | http://silkbase.ab.a.u-tokyo.ac.jp/pub/Bomo_gene_models_prot.fa.gz |
| *Chilo suppressalis* | http://v2.insect-genome.com/api/Download/..-01_data-01_species-Chilo_suppressalis-Chilo_suppressalis.anno.pep.fa |
| *Cnaphalocrocis medinalis* | http://v2.insect-genome.com/api/Download/..-01_data-01_species-Cnaphalocrocis_medinalis-Cnaphalocrocis_medinalis.anno.pep.fa |
| *Spodoptera exigua* | http://v2.insect-genome.com/api/Download/..-01_data-01_species-Spodoptera_exigua-Spodoptera_exigua.anno.pep.fa |
| *Spodoptera frugiperda* | http://v2.insect-genome.com/api/Download/..-01_data-01_species-Spodoptera_frugiperda-Spodoptera_frugiperda.anno.pep.fa |
| *Trichoplusia ni* | http://v2.insect-genome.com/api/Download/..-01_data-01_species-Trichoplusia_ni-Trichoplusia_ni.anno.pep.fa |
| *Spodoptera litura* | https://ftp.ncbi.nlm.nih.gov/genomes/all/GCF/002/706/865/GCF_002706865.1_ASM270686v1/GCF_002706865.1_ASM270686v1_protein.faa.gz |
| *Danaus plexippus* | https://api.ncbi.nlm.nih.gov/datasets/v2alpha/genome/download?filename=GCF_009731565.1.zip&ncbi_phid=322C866E32B3B8B500005EE7B655259B.1.m_1.029 |
| *Melitaea cinxia* | http://ftp.ensemblgenomes.org/pub/metazoa/release-38/fasta/melitaea_cinxia/pep/Melitaea_cinxia.MelCinx1.0.pep.all.fa.gz |
| *Papilio xuthus* | https://ftp.ncbi.nlm.nih.gov/genomes/all/GCF/000/836/235/GCF_000836235.1_Pxut_1.0/GCF_000836235.1_Pxut_1.0_protein.faa.gz |
| *Cydia pomonella* | [http://v2.insect-genome.com/api/Download/..-01_data-01_species-Cydia_pomonella-Cydia_pomonella.anno.pep.fa](http://v2.insect-genome.com/api/Download/..-01_data-01_species-Cydia_pomonella-Cydia_pomonella.anno.pep.fa；http:/v2.insect-genome.com/api/Download/..-01_data-01_species-Cydia_pomonella-Cydia_pomonella.gff3) |
| *Plutella xylostella* | <https://ftp.ncbi.nlm.nih.gov/genomes/all/GCF/000/330/985/GCF_000330985.1_DBM_FJ_V1.1/GCF_000330985.1_DBM_FJ_V1.1_protein.faa.gz> |
| *Drosophila melanogaster* | https://ftp.ensemblgenomes.ebi.ac.uk/pub/metazoa/release-50/fasta/drosophila_melanogaster/pep/Drosophila_melanogaster.BDGP6.32.pep.all.fa.gz |
